# Giant Intracardiac Lipoma: A Case Report and the Role of Multimodality Cardiac Imaging

**DOI:** 10.7759/cureus.29565

**Published:** 2022-09-25

**Authors:** Mutaz Karameh, Mordechai Golomb, Merav Yarkoni, Ehud Rudis, Tal Keidar Haran, Nassem Shadafny, Dotan Cohen, Ronen Beeri, Dan Gilon, Rabea Asleh, Ronen Durst

**Affiliations:** 1 Heart Institute, Hadassah Medical Center, Faculty of Medicine, Hebrew University of Jerusalem, Jerusalem, ISR; 2 Department of Cardiothoracic Surgery, Hadassah Medical Center, Faculty of Medicine, Hebrew University of Jerusalem, Jerusalem, ISR; 3 Department of Pathology, Hadassah Medical Center, Faculty of Medicine, Hebrew University of Jerusalem, Jerusalem, ISR; 4 Department of Radiology, Hadassah Medical Center, Faculty of Medicine, Hebrew University of Jerusalem, Jerusalem, ISR

**Keywords:** cardiac magnetic resonance imaging, left ventricular lipoma, echo cardiogram, cardiology cardiac ct and mri, multimodality cardiac imaging, lipoma

## Abstract

Cardiac lipomas, especially ones originating from the left ventricle, are extremely rare. They may be asymptomatic or may present with various non-specific symptoms. Herein, we report a case of a giant lipoma of the left ventricle, with frequent ventricular premature beats on electrocardiogram. An echocardiogram demonstrated a large hyperechoic mass occupying a significant portion of the left ventricle. We further describe the diagnostic workup utilizing multimodality cardiac imaging and treatment options. Cardiac MRI demonstrated fat suppression, and cardiac CT showed a homogenous low-attenuation mass suggesting lipomatous matter. The mass was subsequently surgically removed for pathology examination in order to rule out liposarcoma. Histopathology demonstrated mature adipocytes, entrapped myocytes with hypertrophy, and interstitial fibrosis foci confirming the diagnosis of lipoma.

## Introduction

Primary cardiac tumors are very rare, with 75% of them being benign. Myxomas are the most common, followed by lipomas and rhabdomyomas. Lipomas are benign neoplasms typically composed of mature fat cells. The reported incidence of primary cardiac lipomas is 0.17-0.19% at autopsy, accounting for 8.4% of all heart and pericardial primary tumors. Cardiac lipoma may involve the endocardium, myocardium, and pericardium [1]. Lipoma should be differentiated from liposarcoma, which is a rare subtype of sarcomas responsible for only 1% of all primary malignant cardiac tumors [1] and carries a poor prognosis and survival rates between weeks to months after diagnosis. The first cases of cardiac primary lipomas and liposarcomas were discovered between the late 16th and mid-19th centuries. Cardiac adipose tissue infiltration is divided into true lipomas, lipomatous hypertrophy of the interatrial septum (LHIS), and lipomatous infiltration. Unlike LHIS, cardiac lipomas are uncommon primary cardiac tumors, and LV lipomas are even rarer.

We report a case of giant intracardiac lipoma originating from the left ventricle (LV) diagnosed by Multimodality cardiac imaging.

## Case presentation

A 38-year-old woman with no past medical history presented to her primary health care due to intermittent back pain for one month. Preliminary workup included an electrocardiogram (ECG), which demonstrated multiple ventricular premature beats, and a transthoracic echocardiogram (TTE), which revealed a cardiac mass in the LV. The patient was referred for further workup and management at our hospital. She reported no other symptoms and denied any relevant personal or familial medical history. Physical examination revealed an occasional grade I/II ejection systolic murmur heard best over the mitral area, and laboratory tests were within normal limits. Repeat ECG showed normal sinus rhythm without signs of arrhythmia or ischemia, and a chest X-ray was unremarkable.

Investigation by multimodality imaging

**Figure 1:**
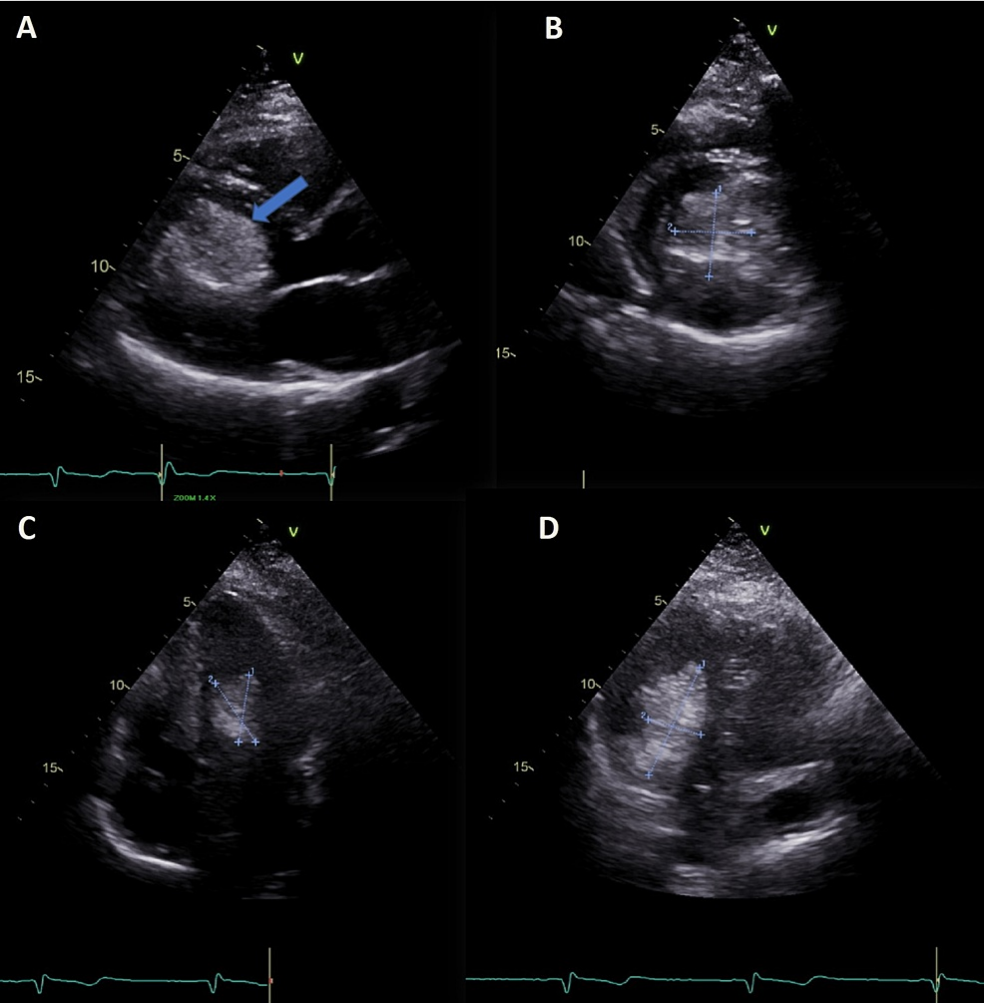
Transthoracic echocardiogram Parasternal long-axis (A), parasternal short-axis (B), four-chamber (C), and three-chamber (D) views showing left-ventricular lipoma (blue arrow).

TTE demonstrated an irregular, large (4.4×3.5 cm) hyperechoic mass occupying a significant portion of the LV (Figure 1 and Videos 1, 2). Color Doppler imaging showed no flow within the mass. LV flow measurement revealed a mild obstruction toward the LV outflow tract (LVOT) with no significant mitral regurgitation.

**Video 1:** Transthoracic echocardiogram Parasternal long-axis view showing left ventricular lipoma.

**Video 2:** Transthoracic echocardiogram Parasternal short-axis view showing left ventricular lipoma.

**Figure 2:**
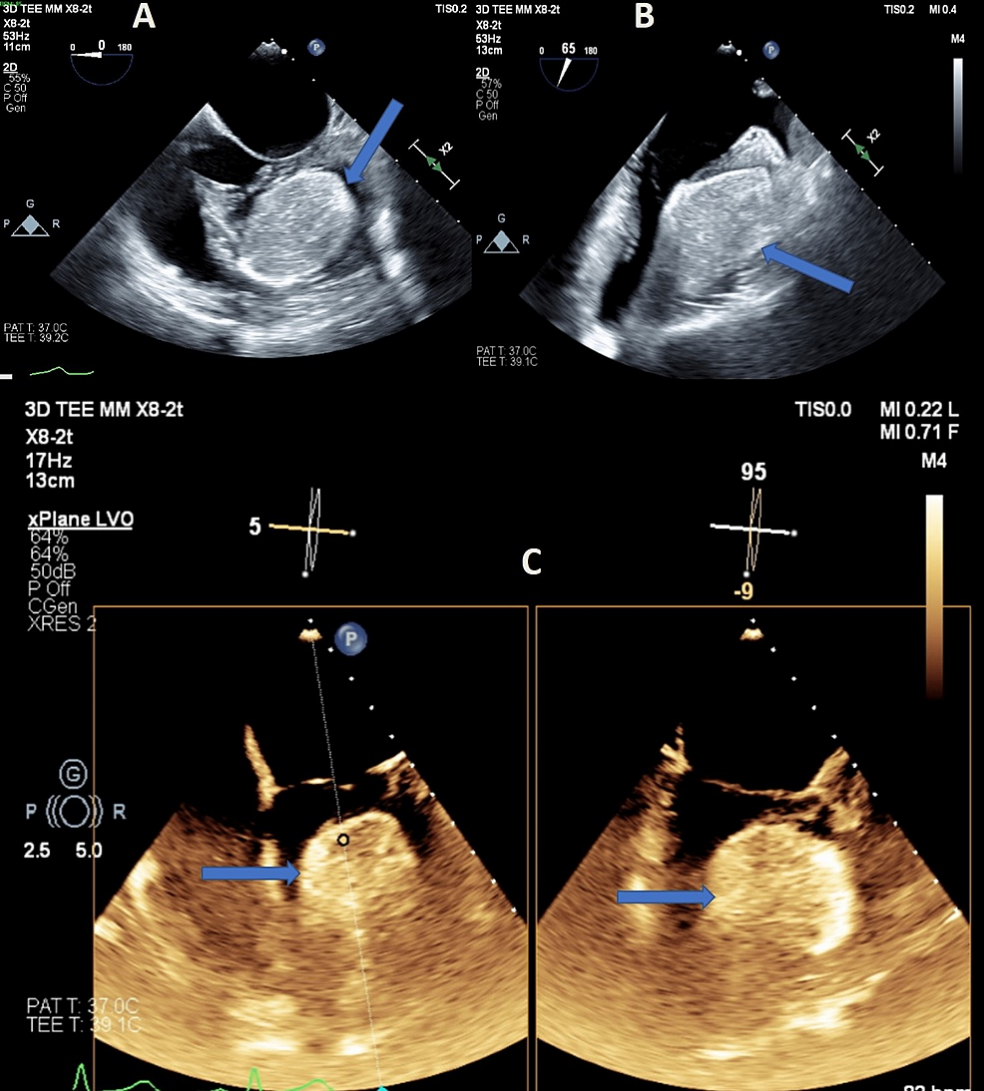
Transesophageal echocardiogram Four-chamber (A) and two-chamber (B) views. (C) Contrast-enhanced four-chamber view with X-plane imaging showing left-ventricular lipoma (blue arrows).

Transesophageal echocardiography (TEE) demonstrated an echogenic mass occupying a significant portion of the LV that was attached to the lateral wall (Figure 2 and Video 3). Both TEE and TTE demonstrated normal ventricular function with preserved ejection fraction and normal valve function.

**Video 3:** Transesophageal echocardiogram Four-chamber view showing a mass in the left ventricle

**Figure 3:**
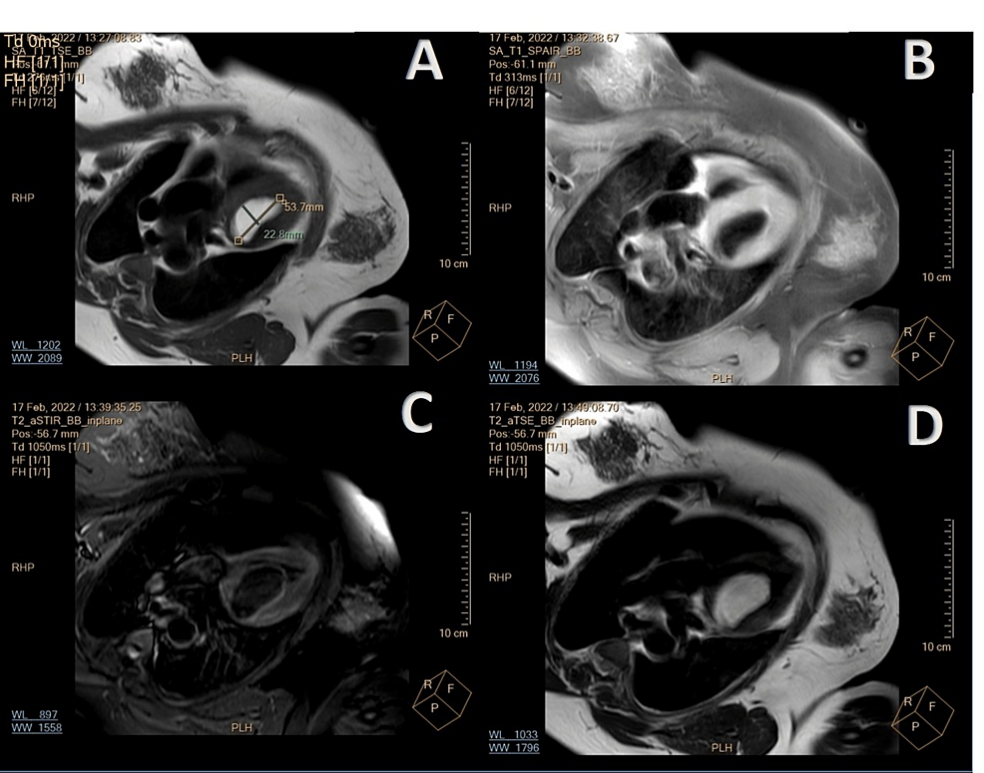
MRI of the mass (A) Panel T1 turbo spin echo (TSE) and (B) TSE with fat saturation. Notice the lack of signal on the fat saturation images. (C)  Panel demonstrates SSFP image of the mass and (D) hyperintense signal of the tumor on T2-weighted images. SSFP, steady-state free precession

Magnetic resonance imaging (MRI) confirmed the presence of an irregular mass in the LV cavity; an oval well-circumscribed mass with sharp and regular margins, measuring 5.7×3.7× 4.3 cm^3^, arising from the anterior and anteroseptal wall, from the LVOT to the mid-ventricular level. This mass was homogeneously hyperintense on T1- and T2-weighted images and completely hypointense on fat-suppressed short T1 inversion recovery (STIR) sequence (Figure 3). The mass showed neither first-pass enhancement nor late gadolinium enhancement. Regional wall motion around the mass was normal.

**Figure 4:**
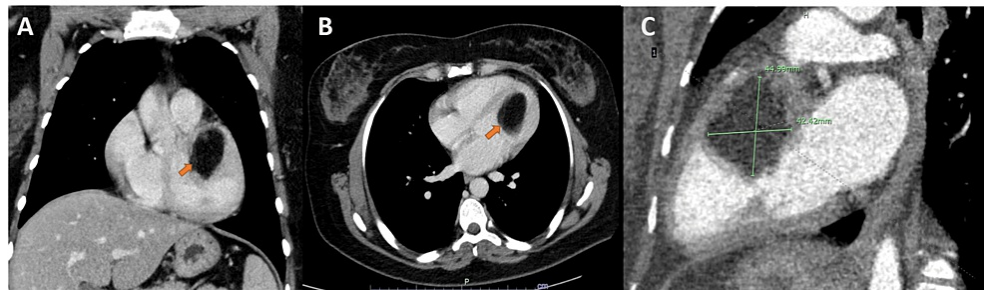
Computed tomography scan Lipoma (orange arrows) on coronal (A), axial (B), and posterior oblique (C) views.

Subsequently, a cardiac gated computerized tomography (CT) examination was carried out with and without the administration of contrast material. Similarly, a low attenuation lipomatous mass was seen on the CT. It was homogeneous, encapsulated, hypodense, and measured 5.5× 3.6× 4.1 cm^3^, arising from the endocardium, projecting into the LV from the anterior, anteroseptal, and anterolateral walls, from the LVOT level to mid ventricle (Figure 4). Cardiac CT showed patent coronaries, and total body CT excluded extracardiac findings.

Management

**Figure 5:**
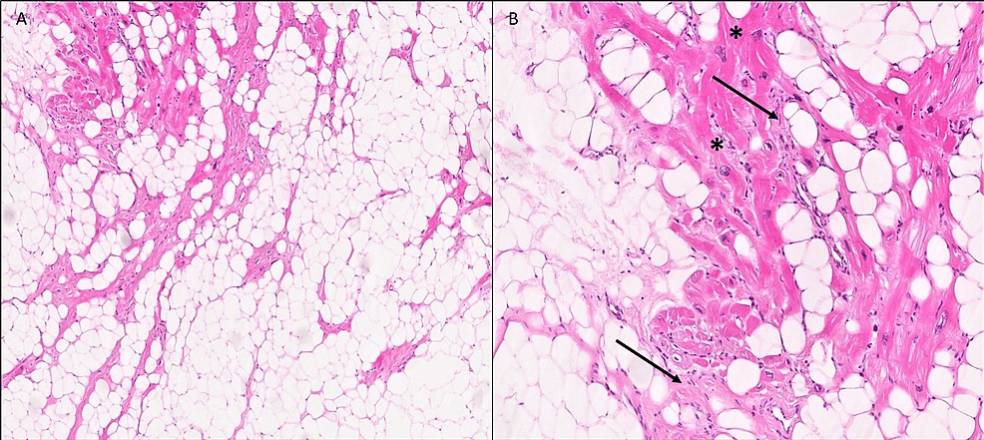
Mass histology Diagnosis of LV lipoma is demonstrated by (A) lipomatous infiltrate on (H&E x4). (B) Entrapped cardiomyocytes show foci of hypertrophy (asterisk) and interstitial fibrosis (arrows) (H&E x20).

After a heart team discussion, the patient underwent surgical removal of the mass, which was yellowish, soft, embedded in the lateral wall of LV, and occupying most of the LV cavity. The mass stalk was not well visualized through the left atriotomy approach; thus, the aortic root approach was used. The tumor encompassed the papillary muscles; therefore, complete removal of the tumor without injuring the valve function was deemed to be nearly impossible. The mass was removed in a piecemeal fashion through the aortic valve. Finally, the mitral and aortic valves were both replaced with bioprosthetic valves. Histopathology demonstrated mature adipocytes, entrapped myocytes with hypertrophy, and interstitial fibrosis foci consistent with a lipoma (Figure 5).

## Discussion

Cardiac lipomas are usually benign and often cause no symptoms. When symptomatic, non-specific symptoms generally relate to mass location, size, and compressive effects on the neighboring cardiac structures causing valvular dysfunction or progressive heart failure, and can also interrupt the conduction system leading to fatal arrhythmias such as ventricular tachycardia [2] or even sudden death [3].

Although, historically, cardiac lipomas were incidental findings during autopsy and cardiothoracic surgery, today non-invasive detection is frequently done by echocardiography, which remains the most convenient non-invasive modality for screening for cardiac masses. However, echocardiography is limited in determining the nature of the mass. Acoustic characteristics of lipomas may exclude cardiac malignancies, especially with contrast media, but differentiation with other benign lesions such as myxoma is difficult [4].

In our case, other imaging methods as CT, and particularly MRI, made cardiac lipoma the most possible diagnosis because of the fatty appearance of a well-prescribed mass. On CT, the lipoma appears as a well-circumscribed homogeneous hypodense, fat attenuated (Hounsfield measurement less than -50 to -150) mass [5]. On MRI, the lipoma appears bright on T1- and T2-weighted images with complete suppression on fat-saturated sequences, which is characteristic of adipose tissue [5].

Although these features are highly specific to cardiac lipoma, it is necessary to differentiate this benign tumor from the highly aggressive liposarcoma. The presence of nodular or globular areas, non-adipose lesions, prominent foci of high T2 signal, the presence of thick septa, or large size mass are suggestive of liposarcoma [6]. MRI is still insufficient for distinguishing between benign lipomas and malignant well-differentiated liposarcomas, which are easily misdiagnosed [7]. The role of positron emission tomography (PET) is well established in evaluating extracardiac lipomas, demonstrating reduced metabolic activity, but is less recognized for evaluating cardiac lipomas [8]. Reported lipomatous hypertrophy containing brown fat may be hypermetabolic on 18-FDG-PET, thus maybe misleading [9]. In a cardiac lesion, due to limited surgical options and the potential for complications, it may be helpful to sample the lesion and, via specific gene markers, reach 94% sensitivity for detecting atypical lipomatous tumors, which will require complete resection [10].

No guidelines for treating cardiac lipomas are currently accepted, and each case is individualized. Symptomatic cardiac lipomas should be resected. Although conservative management may be an alternative option in asymptomatic patients, this is still controversial. Some specialists prefer surgical resection due to potential overgrowth and infiltration into the myocardium, which may lead to unfavorable outcomes. Others suggest clinical follow-up in patients with incidentally noticed small lipomas.

Recurrence of cardiac lipomas after resection is infrequent, with only few reports to date. Reasons for recurrence include incomplete resection due to diffuse infiltration in the myocardium, intraoperative implantation of some parts of the tumor, and multiorigin of the tumor [11]. Resection of the recurrent lipoma was extraordinarily challenging, and heart transplantation may provide the ultimate solution. Long-term echocardiography follow-up should be recommended for these patients [12].

## Conclusions

We reported a case of giant LV lipoma causing back pain and frequent ventricular premature beats. Multimodality cardiac imaging was paramount during the workup; MRI showed fat suppression of the lesion, and CT showed a homogenous low attenuation mass, both suggesting the likely lipomatous nature of the lesion. Since liposarcoma cannot be adequately excluded by imaging, surgical excision was necessary to confirm the diagnosis. Cardiac lipomas, especially LV lipomas, are rare. They may be asymptomatic or may present with various non-specific symptoms. Echocardiography remains the initial diagnostic modality of choice for detecting cardiac lipomas. However, cardiac CT and MRI provide better imaging quality and a better understanding of the cardiac mass and its relation to neighboring structures, further directing care management. Finally, surgical resection remains the gold standard for treating symptomatic cardiac lipomas.
